# Melatonin and Inflammatory Cytokines as Modulators of the Interaction Between Gestational Diabetes Mellitus and Pregnancy-Specific Urinary Incontinence

**DOI:** 10.3390/metabo15110699

**Published:** 2025-10-28

**Authors:** Danielle Cristina Honório França, Eduardo Luzia França, Adenilda Cristina Honório-França, Kênia Maria Rezende Silva, Adriele Ataídes de Queiroz, Tassiane Cristina Morais, Emanuelle Carolina Honorio França, Carolina Neiva Frota de Carvalho, Danny Laura Gomes Fagundes-Triches, Angélica Mércia Pascon Barbosa, Iracema de Mattos Paranhos Calderon, Luis Sobrevia, Marilza Vieira Cunha Rudge

**Affiliations:** 1 Department of Gynecology and Obstetrics, Botucatu Medical School (FMB), São Paulo State University (UNESP), Botucatu 18618-687, Brazil; danielle.franca@unesp.br (D.C.H.F.); carolina.n.carvalho@unesp.br (C.N.F.d.C.);; 2 Institute of Biological and Health Science, Federal University of Mato Grosso (UFMT), Barra do Garças 78605-091, Brazil; eduardo.franca@ufmt.br (E.L.F.);; 3 School of Sciences, Santa Casa de Misericórdia de Vitória (EMESCAM), Vitória 29045-402, Brazil; 4 Department of Physiotherapy and Occupational Therapy, School of Philosophy and Sciences, São Paulo State University (UNESP), Marilia 17525-900, Brazil; 5 Cellular and Molecular Physiology Laboratory (CMPL), Department of Obstetrics, Division of Obstetrics and Gynaecology, School of Medicine, Faculty of Medicine, Pontificia Universidad Católica de Chile, Santiago 8330024, Chile; 6 Department of Physiology, Faculty of Pharmacy, Universidad de Sevilla, E-41012 Seville, Spain; 7 Faculty of Medicine and Biomedical Sciences, University of Queensland, Herston, QLD 4029, Australia

**Keywords:** neuropharmacology, hormones, hyperglycemia, involuntary loss of urine, hormone receptor, immunomodulation, gestation, diabetes

## Abstract

Background: The pathogenesis of developing gestational diabetes mellitus (GDM) integrated with pregnancy-specific urinary incontinence (PSUI) may be related to immunological and hormonal factors. Inflammatory cytokines influence the function and regulation of the urinary tract, and changes in melatonin concentration are a predisposing factor for smooth muscle dysfunction and cystometric changes. Objective: This study examines the influence of melatonin, MT1 and MT2 receptors, and inflammatory cytokines in the blood and urine of pregnant women with GDM and PSUI. Methods: Two hundred sixty-nine pregnant women were approached during the diagnostic investigation of GDM and answered a specifically structured questionnaire about the involuntary loss of urine. According to these criteria, mothers were divided into four groups: continent normoglycemic (NG-C), incontinent normoglycemic (NG-I), continent GDM (GDM-C), and incontinent GDM (GDM-UI). Blood and urine samples were collected to determine the levels of melatonin, melatonin sulfate, melatonin receptors (MT1 and MT2), and inflammatory cytokines. Results: Blood level of melatonin and IL-10 was lower, but MT1, MT2, IL-1β, IL-8, and TNF-α were higher in GDM-UI compared with the NG-C group. The melatonin sulfate level was lower in the urine of the GDM-UI group compared with the NG-C group. Conclusions: Maternal hyperglycemia associated with urinary incontinence generates an inflammatory environment characterized by reduced melatonin and IL-10 and increased IL-1β, IL-8, and TNF-α in the blood of mothers with GDM with UI. This environmental condition may be involved in the pathogenesis of these pathologies.

## 1. Introduction

Pregnancy is associated with metabolic changes that involve the increased release of insulin counter-regulatory hormones, leading to insulin resistance and glucose intolerance, which can contribute to the development of Gestational Diabetes Mellitus (GDM) [1]. This condition impacts maternal and neonatal health worldwide, increases health care costs, and predisposes to maternal–fetal complications [2,3]. Women with GDM are at higher risk of developing pregnancy-specific urinary incontinence (UI) [4], a condition that significantly affects biopsychosocial aspects of pregnancy. Hyperglycemia has been associated with increased relaxation of pelvic floor muscles [5], while insulin resistance contributes to muscle atrophy and weakness, both of which predispose to urinary incontinence [6,7].

The hyperglycemic maternal microenvironment in GDM is also associated with inflammation, as it releases proinflammatory cytokines [8]. These molecules regulate inflammatory responses, act in the brain and spinal cord, influence micturition, and have peripheral effects on bladder function. Oxidative stress and inflammation are also key mediators in the onset and progression of smooth muscle dysfunctions [9].

Genetic and hormonal factors and maternal eating habits are also involved in GDM and PSUI. Systemic inflammation modulates the uptake of molecules and directly influences the production of hormones, such as melatonin [10,11]. Melatonin is the immunomodulatory agent mainly produced and secreted by the pineal gland during its nocturnal peak [11,12,13], being directly influenced by the light diffused through the retina to the hypothalamic suprachiasmatic nucleus in the Central Nervous System (CNS) [10].

Melatonin has been related to the regulation of the circadian cycle and rhythmically participates in the modulation of the secretion of insulin and glucose molecules [11], favors the differentiation of neurons, and has the anti-apoptotic and antioxidative potential [14,15], being excreted in the form of its metabolite melatonin–sulfate (6-Sulfatoxymelatonin) in the urine [16].

This molecule also exerts protective effects on smooth muscle physiology by restoring detrusor contractility, normalizing mitochondrial function, and modulating neuromuscular activity, thereby preventing cystometric changes and inflammation [15]. Its biological actions are mediated by MT1 and MT2 receptors, expressed not only in the CNS but also in peripheral tissues, which synchronize circadian rhythm and metabolic functions [17,18,19]. Imbalance in this signaling system is directly linked to the pathophysiology of diabetes and related complications [20].

An imbalance in the biological actions of this hormone is linked to the pathophysiology of metabolic diseases, including diabetes mellitus [20]. However, the roles of inflammatory regulatory cytokines and melatonin in maintaining urinary continence in pregnant women with GDM are still unclear. This study evaluated the levels of melatonin, MT1 and MT2 receptors, and inflammatory cytokines in the blood and urine of pregnant women with GDM and PSUI.

## 2. Materials and Methods

### 2.1. Study Population

This study is a cross-sectional observational study conducted at the Perinatal Diabetes Research Center at the Botucatu Medical School (FMB—UNESP, Brazil). Pregnant women who met the following inclusion criteria were included: age between 18 and 40 years, GDM diagnosis, and a single pregnancy with a live fetus. Patients signed the Informed Consent Form. Pregnant women with urinary incontinence before pregnancy, Type 1 or Type 2 diabetes mellitus, pregnant women with polycystic ovary syndrome, or conception by assisted reproduction were excluded from the study. Multiple pregnancies, collagen diseases, autoimmune diseases, use of tricyclic antidepressants, and fetal malformations were also excluded from the study.

Two hundred sixty-nine pregnant women were approached during the GDM diagnostic investigation, which included the 75-g oral glucose tolerance test (OGTT), fasting glucose, and glycemic profile. The diagnosis of gestational diabetes mellitus (GDM) was established according to the American Diabetes Association [21] criteria, based on plasma glucose. The participants answered the International Consultation on Incontinence Questionnaire—Short Form (ICIQ-SF) [22], a questionnaire validated in Brazil that assesses the impact of UI on women’s quality of life to classify urinary loss and determine the severity index of UI [23]. The questionnaire was validated as a quantitative and semi-objective measure of UI severity.

**Figure 1:**
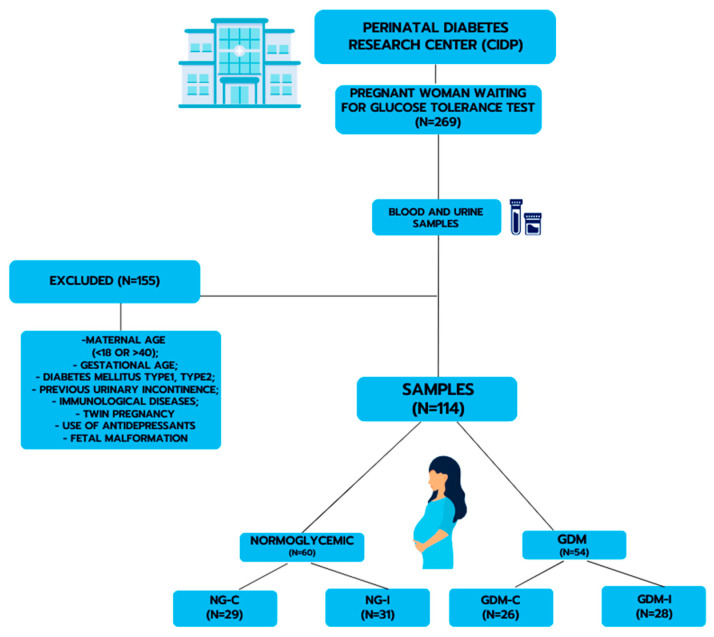
Representative scheme for obtaining samples and experimental design.

Following the selection criteria of the participants, 114 pregnant women were included in the study and classified into the following study groups: continent normoglycemic (NG-C, 29 patients), normoglycemic with pregnancy-specific urinary incontinence (NG-I, 31 patients), continent with GDM (GDM-C, 26 patients), and GDM with pregnancy-specific urinary incontinence (GDM-I, 28 patients). The scheme for obtaining samples and experimental design is described in Figure 1. All patients signed the Informed Consent Form.

### 2.2. Blood and Urine Samples

Blood samples (8 mL) were collected in Vacutainer^®^ tubes (Becton Dickinson, Franklin Lakes, NJ, USA) without anticoagulant and centrifuged (160× *g*, 15 min, at room temperature). The serum was separated and stored at −80 °C for further analysis. The leukocytes were separated by density gradient and lysed with Triton X-100 (Sigma, St. Louis, MO, USA) for 5 min at room temperature. The lysate was centrifuged (1500× *g*, 10 min, 8 °C) to remove cellular debris.

Urine samples (15 mL) were collected in the morning, as the first voids of the day, to reduce circadian variability in melatonin metabolite levels. During a period of moderate urinary flow, samples were collected in sterile urine containers in accordance with aseptic collection guidelines. Subsequently, urine samples were centrifuged (160× *g*, 15 min, at room temperature), and supernatants were stored at −80 °C for later analysis.

### 2.3. Melatonin Determination

Quantitative determination of melatonin hormone concentration in human serum was performed by enzyme immunoassay on ELISA microplates with an ELISA kit (Imuno-Biological Laboratories-Melatonin-ELISA RE54021-IBL, Hamburg, Germany). The lower detection limit was 1.6 pg/mL, with intra-assay and inter-assay coefficients of variation (CV%) of 3.0% to 11.4% and 6.4% to 19.3%, respectively. The melatonin was extracted by affinity chromatography and concentrated in a vacuum centrifuge (500× *g*, 180 min, 4 °C). Reaction was measured by absorbance at 450 nm in a plate spectrophotometer (ThermoPlate TP-Reader, Thermo Fisher Scientific, Waltham, MA, USA). The results were calculated against a melatonin standard curve and expressed in picograms per milliliter (pg/mL).

### 2.4. Melatonin Receptors Determination

A sandwich enzyme immunoassay ELISA kit (Cloud Clone Corporation, Houston, TX, USA) was used for in vitro quantitative measurement of melatonin receptor 1A (MT1) and 1B (MT2) in lysed human blood cells according to the manufacturer’s instructions. The lower detection limit was 156 pg/mL (0.156 ng/mL), and intra-assay and inter-assay CV% were <10% and <12%, respectively. Reaction was measured by absorbance at 450 nm in a plate spectrophotometer (Thermo Plate TP-Reader, Thermo Fisher Scientific, Waltham, MA, USA). The results were calculated against MT1 and MT2 standard curves and expressed as picograms per milliliter (pg/mL).

### 2.5. Urinary Melatonin–Sulfate Measurement

The concentration of melatonin–sulfate urine (sMT6a) was measured by enzyme immunoassay on microplates using an ELISA kit (Imuno-Biological Laboratories–Melatonin–sulfate Urine ELISA RE54031-IBL, Hamburg, Germany). The lower detection limit was 1.0 ng/mL, and the intra-assay and inter-assay coefficients of variation (CV%) were 5.2% to 12.2% and 5.1% to 14.9%, respectively. Reaction was measured by absorbance at 450 nm in a plate spectrophotometer (ThermoPlate TP-Reader, Thermo Fisher Scientific, Waltham, MA, USA). The results were calculated against a melatonin sulfate standard curve and expressed in nanograms per milliliter (ng/mL). For the melatonin/melatonin–sulfate ratio, the results of melatonin–sulfate were expressed in pg/mL.

### 2.6. Cytokines Determination

Cytokine concentrations in serum and urine samples were evaluated using the Cytometric Bead Array (CBA) Kit (BD Bioscience, Franklin Lakes, NJ, USA) according to the manufacturer’s instructions. The cytokines IL-1β, IL-6, IL-8, IL-10, IL-12, and TNF-α were analyzed using flow cytometry (FACS Calibur, BD Bioscience, USA). The results were generated using the BD CellQuest Pro Software Acquisition (version 5.1, BD Biosciences, USA) and analyzed using the FCAP Array Software v3.0 (BD Biosciences, San Jose, CA, USA).

### 2.7. Statistics

Statistical analyses were performed using BioEstat^®^ version 5.0 software (Mamirauá Institute, Belém, Brazil). Data were expressed as means ± standard deviations (SD). The sample size was estimated using the same software, assuming a statistical power of 80% (β = 0.20) and a significance level of 5% (α = 0.05). The calculation was based on previous studies from our research group that evaluated similar immunometabolic parameters and indicated an expected difference of approximately 20% between groups.

The D’Agostino normality test was applied to assess data distribution. Two-way analysis of variance (ANOVA) followed by the Bonferroni post-test was used for group comparisons. Pearson’s correlation test was applied to evaluate associations between cytokine levels, melatonin concentrations, and metabolic parameters. The adopted significance criterion was *p* < 0.05.

Additionally, Cohen’s d was used to estimate the magnitude of intergroup differences, and results were interpreted according to conventional thresholds: d = 0.2 (small), d = 0.5 (medium), and d ≥ 0.8 (large).

## 3. Results

### 3.1. Study Groups

The clinical information of the mothers is given in Table 1. Mothers with GDM, regardless of the presence or absence of urinary incontinence, were older than those in the corresponding normoglycemic groups. The GDM groups had higher BMI and glycaemia (*p* < 0.05), and the GDM-UI group had a higher HbA1c level than all other groups. However, no significant difference in urine density was observed between the groups.

### 3.2. Blood Melatonin and Melatonin Receptors

**Figure 2:**
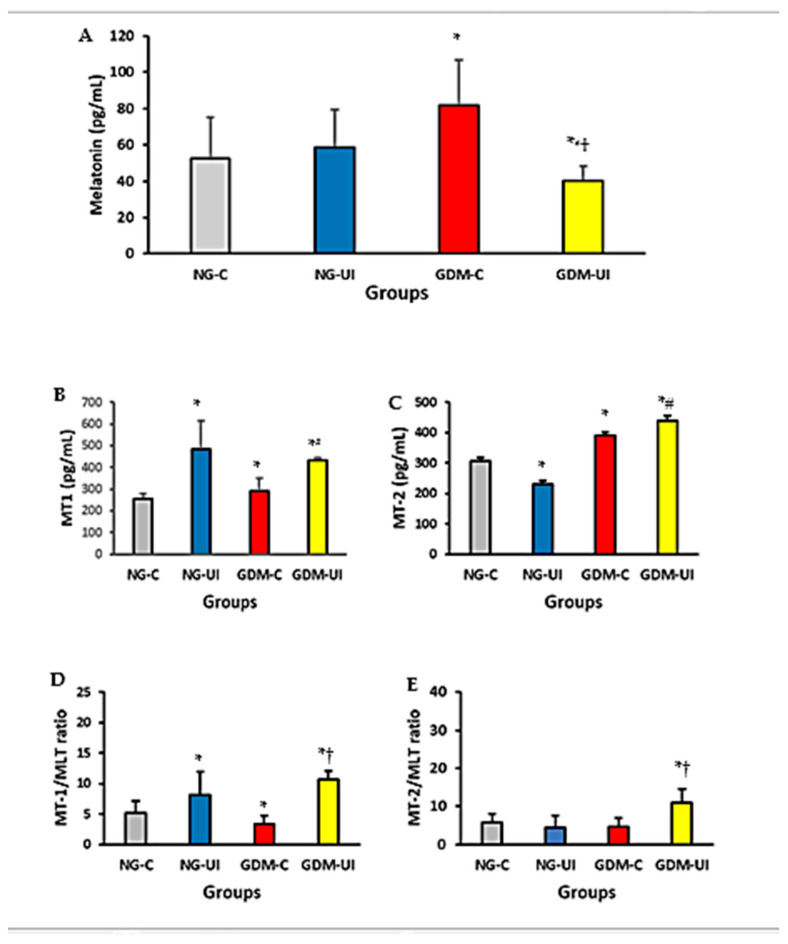
Blood levels of melatonin and its receptors in pregnant women. (**A**) Melatonin concentration; (**B**) MT1 receptor level; (**C**) MT2 receptor level; (**D**) MT1/melatonin (MT1/MLT) ratio; (**E**) MT2/melatonin (MT2/MLT) ratio. Measurements were performed in women with normoglycemia (NG) or gestational diabetes mellitus (GDM), without urinary incontinence (NG-C, GDM-C) or with urinary incontinence (NG-UI, GDM-UI). Data are presented as mean ± standard error of the mean (S.E.M.). Significant differences between normoglycemic and GDM groups were observed for melatonin (*p* = 0.0268), MT1 (*p* = 0.0394), MT2 (*p* = 0.0205), MLT/MT1 (*p* = 0.0066), and MLT/MT2 (*p* = 0.0205). Symbols of significance: * difference between the control group (NG-C) and the other groups (NG-UI, GDM-C, and GDM-UI); # difference between NG-UI and GDM-UI groups; †—difference between GDM-C and GDM-UI (*p* < 0.05 vs. GDM-C).

The levels of melatonin, as well as those of the MT1 and MT2 receptors, were measured in blood samples from pregnant women. The serum level of melatonin was higher in GDM-C but lower in GDM-UI groups compared with NG-C or NG-UI groups (Figure 2A). The melatonin serum level was lower in the GDM-UI group compared with the GDM-C group.

The MT1 receptor level in the blood of NG-UI and GDM-UI groups was higher than in NG-C and GDM-C groups (Figure 2B). The MT2 receptor level was higher in the blood of GDM-C and GDM-UI but lower in the NG-UI groups compared with the NG-C group (Figure 2C). Additionally, the MT2 receptor blood level in the NG-UI group was lower than in the GDM-C and GDM-UI groups.

The MT1/melatonin ratio was higher in NG-UI and GDM-UI but lower in GDM-C compared with NG-C groups (Figure 2D). The MT1/melatonin ratio in GDM-UI was also higher than that of the GMD-C group. However, the MT2/melatonin ratio was higher in the GDM-UI group compared with all other groups in this study.

**Figure 3:**
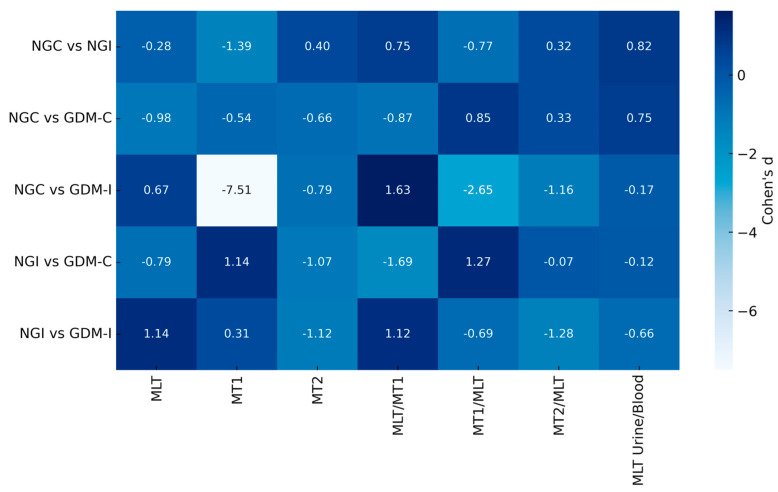
Heatmap of Effect Sizes (Cohen’s d) for melatonin and its receptors. Heatmap representing the effect sizes (Cohen’s d) of group comparisons for circulating and their receptors. Each square indicates the magnitude and direction of the difference between groups (NG-C, NG-UI, GDM-C, and GDM-UI). Lighter colors denote higher effect sizes, illustrating the most pronounced differences observed in MT1 and MLT/MT1 markers, particularly in the GDMI group.

Serum melatonin and its MT1 and MT2 receptors demonstrated distinct modulation patterns between groups (Figure 3). Effect size analysis revealed that MLT concentrations were moderately increased in GDM-C (Cohen’s d = −0.98 vs NGC) but reduced in GDM-UI (Cohen’s d = 0.67 vs NG-C). For the receptor, the most pronounced difference was observed in MT1 (Cohen’s d = −7.51, NG-C vs GDM-UI).

### 3.3. Urine Melatonin–Sulfate Level

**Figure 4:**
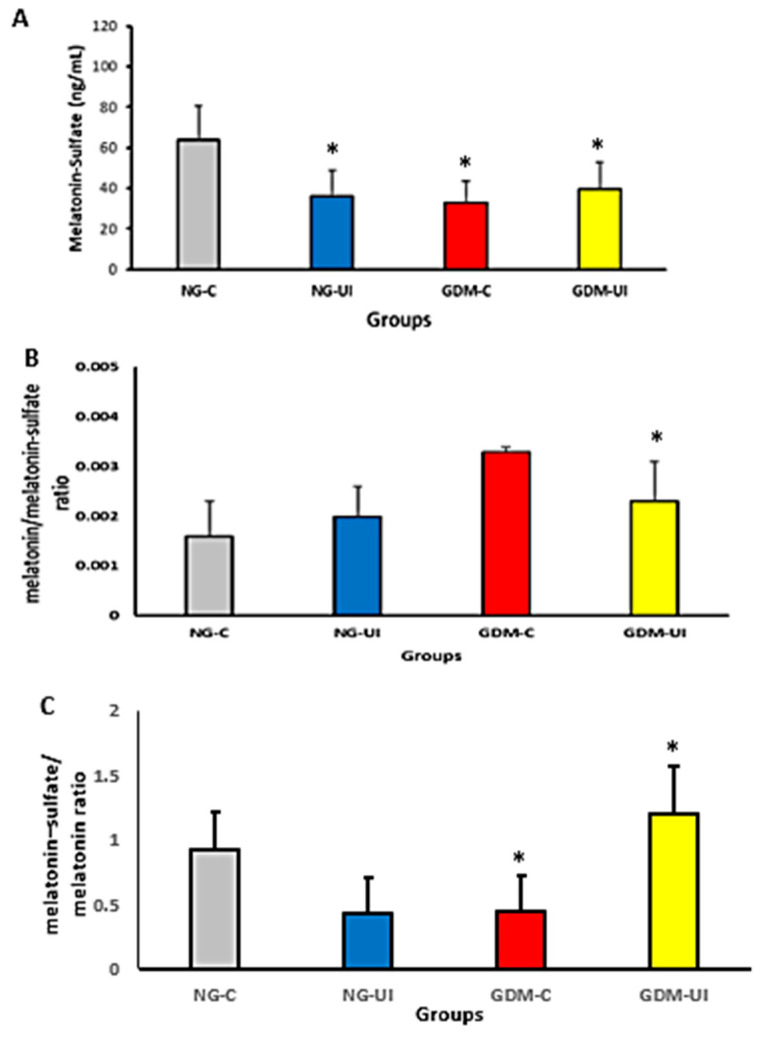
Dynamics of Urinary and Circulating Melatonin in Women with and without Gestational Diabetes Mellitus and Urinary Incontinence. (**A**) Urinary melatonin–sulfate concentration (MLT-S). (**B**) Serum melatonin/urinary melatonin–sulfate ratio (MLT/MLT-S) and (**C**) Urine melatonin–sulfate/melatonin serum ratio (MLT) by group, calculated from measured data. Groups are color-coded as NG-C (gray), NG-UI (blue), GDM-C (red), and GDM-UI (yellow). Measurements were performed in women with normoglycemia (NG) or gestational diabetes mellitus (GDM), without urinary incontinence (NG-C, GDM-C) or with urinary incontinence (NG-UI, GDM-UI). Data are expressed as mean ± standard error of the mean (S.E.M.). Significant differences between normoglycemic and GDM groups were observed for melatonin–sulfate (*p* = 0.0093), MLT serum/MLT urine (*p* = 0.0153), and MLT urine/MLT serum (*p* = 0.0048). Symbols of significance: * *p* < 0.05 vs. control group (NG-C).

Melatonin–sulfate levels were measured in urine samples from pregnant women. Urinary melatonin–sulfate concentrations were lower in NG-UI, GDM-C, and GDM-UI groups compared with the normoglycemic continent group (NG-C) (Figure 4A). The blood melatonin-to-urine melatonin–sulfate ratio was higher in GDM-C compared with all other groups (Figure 4B). Conversely, the inverse ratio (urine melatonin–sulfate to blood melatonin) was lower in NG-UI and GDM-C and elevated in GDM-UI (Figure 4C).

Effect size analysis supported these findings, indicating a moderate difference between GDM-C and NG-C (Cohen’s *d* = 0.63), a small effect for GDM-UI (*d* = 0.18), a large difference between NG-C and NG-UI (*d* = 0.82), and a moderate difference between NG-UI and GDM-UI (*d* = −0.66).

### 3.4. Serum and Urine Cytokine Levels

**Figure 5:**
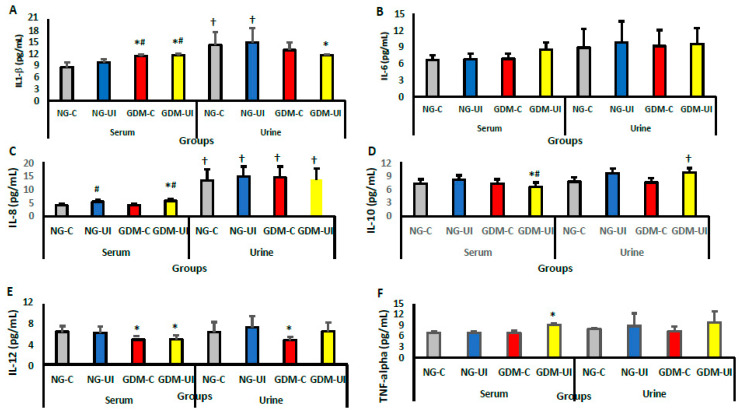
Cytokine concentrations (pg/mL) measured in serum and urine samples. (**A**) IL-1β; (**B**) IL-6; (**C**) IL-8; (**D**) IL-10; (**E**) IL-12; (**F**) TNF-α. Analyses were conducted in women with normoglycemia or gestational diabetes mellitus (GDM), classified as continent or with urinary incontinence (UI). Data are presented as mean ± standard error of the mean (S.E.M.). Group abbreviations: NG-C, normoglycemic continent; NG-UI, normoglycemic with urinary incontinence; GDM-C, GDM continent; GDM-UI, GDM with urinary incontinence. Significant differences with corresponding *p*-values were observed as follows: IL-1β (*p* = 0.0010), IL-6 (*p* = 0.6241), IL-8 (*p* = 0.0484), IL-10 (*p* = 0.0120), IL-12 (*p* = 0.0229), and TNF-α (*p* < 0.0001) between normoglycemic and GDM groups (*); IL-1β (*p* = 0.0001), IL-6 (*p* = 0.3994), IL-8 (*p* = 0.0231), IL-10 (*p* = 0.0416), IL-12 (*p* = 0.5645), and TNF-α (*p* = 0.1181) between continent and incontinent groups (#); and IL-1β (*p* = 0.0001), IL-6 (*p* = 0.5888), IL-8 (*p* = 0.0308), IL-10 (*p* = 0.0450), IL-12 (*p* = 0.8397), and TNF-α (*p* = 0.3035) between serum and urine samples (†). Symbols: * normoglycemic vs. GDM; # continent vs. incontinent; † serum vs. urine.

**Figure 6:**
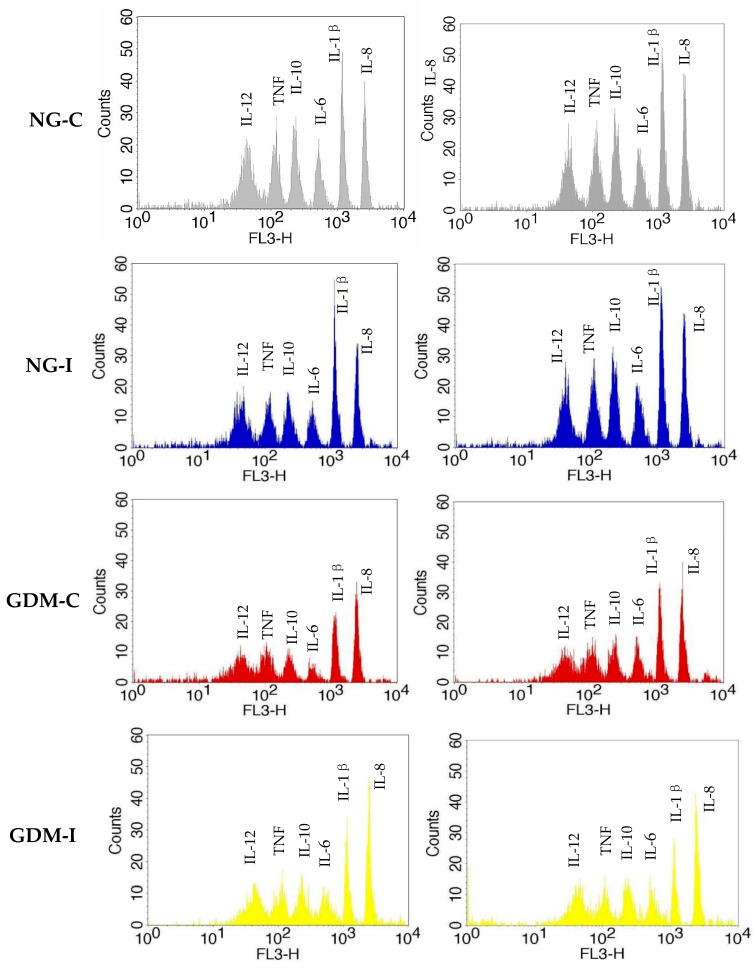
Fluorescence intensity of cytokines in serum and urine. Fluorescence analyses were performed using flow cytometry (FACSCalibur, Becton Dickinson, Franklin Lakes, New Jersey, USA). Cytokine levels were quantified as fluorescence intensity peaks (FL3 channel). Measurements were performed in women with normoglycemia or gestational diabetes mellitus (GDM), without urinary incontinence (NG-C, GDM-C) or with urinary incontinence (NG-UI, GDM-UI).

The levels of IL-1β, IL-6, IL-8, IL-10, IL-12, and TNF-α cytokines were assessed in the serum and urine (Figure 5 and Figure 6). The serum level of IL-1β was higher in GDM-C and GDM-IU compared with NG-C and NG-UI groups (Figure 5A). However, the level of this cytokine in the urine remained unchanged. The IL-1β level in serum was lower compared with urine only in the NG-C and NG-UI groups. The IL-6 levels were similar across all experimental groups (Figure 5B). The serum level of IL-8 was higher in the NG-UI and GDM-UI groups than in the NG-C and GDM-C groups; however, the urine level of this cytokine was unchanged between the groups (Figure 5C). All IL-8 concentrations detected in the urine were higher than those in blood samples in all patient groups.

The serum level of IL-10 was lower in GDM-UI compared with all other groups in both serum and urine (Figure 5D). IL-12 serum levels in GDM-C and GDM-UI were lower than in the NG groups (Figure 5E). However, the level of TNF-α in serum was higher in GDM-UI than in all other groups, both in serum and in urine (Figure 5F).

The serum/urine ratio for IL-1β was lower in NG-C, and the ratio for IL-12 was higher in GDM-UI (Table 2). However, the serum-to-urine ratios for IL-6, IL-8, IL-10, and TNF-α were similar between the groups (Table 2). Changes in blood melatonin levels were positively correlated with IL-10 but negatively correlated with TNF-α levels (Table 3). No significant correlation was found between blood melatonin and the other cytokines. Additionally, changes in urinary melatonin–sulfate levels were positively correlated with IL-6 and IL-10 levels (Table 4). No significant correlation was found between urinary melatonin sulfate levels and the other cytokines.

## 4. Discussion

Gestational diabetes mellitus (GDM) is a pregnancy-related metabolic disorder characterized by hyperglycemia, which can compromise the structural integrity and contractile function of pelvic and abdominal muscles through mechanisms involving insulin resistance and altered glucose metabolism. These alterations may lead to muscle relaxation, weakness, and atrophy, ultimately predisposing women to the development of pregnancy-specific urinary incontinence (PSUI) [4,6,24]. Since GDM is associated with increased release of proinflammatory cytokines [8], and systemic inflammation increases melatonin synthesis [25], this study aimed to determine whether women with GDM and PSUI showed altered blood and urine levels of melatonin and inflammatory cytokines. The findings suggest that women with GDM who develop PSUI show an altered pattern of cytokines in the blood and urine in which melatonin may play a role.

Various risk factors associated with GDM include pre-pregnancy body mass index (BMI) and gestational weight gain (GWG). The women with GDM included in this study showed pre-pregnancy BMI higher than in normal pregnancies, agreeing with other reports in patients with this disease [26,27,28]. Women in the GDM-UI group had the highest BMI between 24 and 28 weeks of gestation, supporting the possibility that these women may be more likely to develop GDM-associated urinary incontinence due to increased weight, compared with women with normal or overweight BMI [29].

Several hormonal factors play key roles in response to hyperglycemia in pregnancy. Pregnant women with type 2 diabetes mellitus (T2DM) showed elevated serum and colostrum levels of melatonin [12,30]. The latter might be a defense mechanism triggered in pregnant women with pre-established diabetes since melatonin acts as a protective factor against smooth muscle disorders [31]. To date, melatonin has been shown to restore the impaired contraction activity of the pelvic floor muscles in patients with urinary incontinence, attenuate sympathetic tone, improve neuromuscular function, protect against inflammation, and scavenge free radicals [15,32,33]. Our findings indicate that serum melatonin concentrations were higher in pregnant women with GDM and urinary continence (GDM-C) compared with those with GDM-UI. This elevation may represent a compensatory mechanism to maintain homeostasis in a hyperglycemic environment [28]. Conversely, women with GDM-UI exhibited reduced melatonin levels, suggesting a loss of this protective effect. Women with urinary incontinence, regardless of glycemic status, also showed lower urinary melatonin–sulfate concentrations, consistent with altered melatonin metabolism.

Changes in the pressure–volume dynamics of the bladder can disrupt neuro-muscular and smooth muscle regulation, leading to impaired detrusor contractility [33,34]. These abnormalities have been shown to improve following melatonin treatment, reinforcing the neuroendocrine role of melatonin in urinary control [35,36]. The hormone acts primarily through its receptors MT1 and MT2, whose deficiency has been associated with insulin resistance and reduced glycolytic activity, respectively [37,38]. Despite lower circulating melatonin in GDM-UI, MT1 receptor expression was increased in leukocytes, suggesting receptor upregulation as a compensatory response.

Regarding the MT2 receptor, there was an increase in both diabetic groups. This increase in receptors, associated with the lower melatonin concentration in GDM-UI pregnant women, demonstrates that, despite the availability of receptors to transmit neuroendocrine signals, the decrease in melatonin probably reflects difficulty in improving detrusor activity in pregnant women with GDM. In addition, the NG-UI group had lower levels of MT2, suggesting that urinary incontinence may predispose to the worsening of myoneural signaling involved in the hormone mechanism melatonin during pregnancy.

Effect size analysis corroborated these observations, revealing large differences between NG-C and NG-UI and moderate divergence between NG-UI and GDM-UI, consistent with a progressive disruption of the pineal–renal feedback axis. Taken together, these findings suggest that GDM-C retains circulating melatonin due to reduced renal elimination. In contrast, GDM-UI exhibits increased urinary loss despite lower serum levels, reflecting inefficient renal conservation. Physiological pregnancy (NG-C) maintains a balanced melatonin profile, while urinary incontinence, particularly in the context of gestational diabetes, appears to alter melatonin homeostasis and receptor expression.

It is reported that mothers who experienced GDM have endocrine and immune system changes [15]. Melatonin is an immunomodulatory agent during pregnancy due to its antioxidant effects and its potential to regulate processes involved in human reproduction [39], primarily by modulating cytokine synthesis and release [40]. Proinflammatory cytokines, such as IL-1β, IL-6, IL-12, IL-8, and TNF-α, regulate micturition at the spinal cord and brain levels, leading to afferent nerve hypersensitivity and promoting hypertrophy of the bladder muscle [41]. In the present study, women in the GDM-UI group showed increased serum levels of IL-8 and TNF-α but reduced levels of the anti-inflammatory cytokine IL-10. These results suggest a proinflammatory profile that contributes to the pathophysiology of GDM associated with PSUI. However, TNF-α may be embryotoxic when increased in serum and tissue in diabetes mellitus [42]. Therefore, the increase in serum TNF-α in women with GDM-UI may be detrimental to the maternal-fetal binomial.

Interestingly, urinary cytokine profiles have been studied in other urinary tract disorders, such as bladder pain syndrome, as potential biomarkers of the pathology [43]. However, the involvement of cytokines in the etiology of GDM-associated urinary incontinence is unknown. The results show that melatonin and cytokine levels in urine and blood were correlated only for women with GDM-UI. The reduced melatonin levels observed in this group of pregnant women correlated positively with IL-10 and inversely with TNF-α, reinforcing the possibility that urinary incontinence may result from a defective biological effect of melatonin. Reduced urinary melatonin sulfate levels in women with GDM-UI showed a positive correlation with IL-10 and melatonin, suggesting that hyperglycemia and urinary incontinence alter endocrine and immune systems, creating an inflammatory environment in women with GDM and pregnancy-specific urinary incontinence.

A limitation of this study is its cross-sectional design, which does not allow for inferences about the temporal or mechanistic relationships between gestational diabetes mellitus, pregnancy-specific urinary incontinence, melatonin levels, and cytokine profiles. Furthermore, maternal age, body mass index (BMI), and gestational age differed between groups, which may have influenced cytokine and melatonin levels; consequently, only associations can be observed. Future longitudinal studies are needed to clarify the effects of these variables and the underlying mechanisms involved.

Despite these limitations, this study has several strengths. The assessment of melatonin levels, its receptors (MT1 and MT2), and inflammatory cytokines in serum and urine provides an integrative view of neuroimmunoendocrine interactions during pregnancy. Assessment of the systemic and excretory compartments identified potential regulatory patterns in melatonin metabolism associated with gestational diabetes and urinary incontinence. This comprehensive approach represents a methodological advantage and contributes new evidence supporting the involvement of the melatonin system in pregnancy-related metabolic and inflammatory conditions.

## 5. Conclusions

Maternal hyperglycemia associated with urinary incontinence creates an inflammatory environment, characterized by reduced melatonin and IL-10 and increased IL-1β, IL-8, and TNF-α in the plasma of mothers with gestational diabetes mellitus and incontinence-specific urinary tract issues. These changes act as modulators in the pathogenesis of both pathologies.

## Figures and Tables

**Table 1:** Clinical data on pregnant women.

	NG-C (n = 29)	NG-UI (n = 31)	GDM-C (n = 26)	GDM-UI (n = 28)
Age	25.0 ± 4.6	27.1 ± 7.2	30.5 ± 6.1 *	30.9 ± 6.3 *†
BMI	29.0 ± 4.2	30.0 ± 5.5	34.7 ± 8.2 *	36.0 ± 7.1 *†
Glucose (mg/dL)	76.2 ± 6.9	76.2 ± 7.9	93.2 ± 10.6 *	92.0 ± 10.3 *†
HbA1c (%)	4.9 ± 0.3	5.0 ± 0.3	5.0 ± 0.4	5.2 ± 0.4 *†
Urinary density	1.0 ± 0.1	1.01 ± 0.1	1.0 ± 0.1	1.2 ± 0.1

Note: NG-C: normoglycemic continent, NG-UI: normoglycemic with urinary incontinence, GDM-C: gestational diabetes mellitus continent, and GDM-UI: gestational diabetes mellitus with urinary incontinence; BMI at 24 to 28 weeks of pregnancy; HbA1c, glycated hemoglobin; PSUI, pregnancy-specific urinary incontinence. The age, BMI, Glycemia, HbA1c, and urinary density are expressed as mean ± S.E.M. * *p* < 0.05 versus NG-C and NG-UI. † *p* < 0.05 versus NG-UI.

**Table 2:** Plasma/Urine cytokine ratio.

Cytokines	Blood/Urine Ratio				Statistical
	NG-C	NG-UI	GDM-C	GDM-UI	
IL-1 β	0.63 ± 0.14	0.88 ± 0.31 *	0.89 ± 0.10 *	1.06 ± 0.04 *	F = 17.6607;
					*p* = 0.0001
IL-6	0.86 ± 0.32	0.92 ± 0. 50	0.80 ± 0.30	0.86 ± 0.28	F = 0.1333;
					*p* = 0.9387
IL-8	0.39 ± 0.12	0.38 ± 0.11	0.37 ± 0.09	0.46 ± 0.22	F = 0.3809;
					*p* = 0.7704
IL-10	0.97 ± 0.27	0.90 ± 0.22	0.96 ± 0.15	0.87 ± 0.22	F = 0.4176;
					*p* = 0.7450
IL-12	1.01 ± 0.41	0.99 ± 0.34	0.94 ± 0.22	1.20 ± 0.15 *	F = 2.3077
					*p* = 0.0482
TNF-α	0.86 ± 0.05	0.85 ± 0.18	0.98 ± 0.15	0.90 ± 0.33	F = 1.2564;
					*p* = 0.3088

Note: Results are expressed as the mean plasma/urine ratio. * Statistical difference between ND-C and the other groups.

**Table 3:** Correlation between plasma melatonin and cytokines.

Melatonin	NG-C	NG-UI	GDM-C	GDM-UI
IL-1 β	r = −0.4102 *p* = 0.3607	r = −0.6301 *p* = 0.1292	r = −0.6414 *p* = 0.1204	r = 0.2530 *p* = 0.5840
IL-6	r = 0.0710 *p* = 0.8797	r = 0.0690 *p* = 0.8831	r = −0.2908 *p* = 0.5269	r = 0.2798 *p* = 0.5433
IL-8	r = 0.4632 *p* = 0.2951	r = 0.2298 *p* = 0.6200	r = 0.2911 *p* = 0.5265	r = 0.0346 *p* = 0.9413
IL-10	r = 0.2824 *p* = 0.5394	r = −0.2685 *p* = 0.5605	r = 0.5486 *p* = 0.2021	r = 0.8980 * p = 0.0470
IL-12	r = −0.4874 *p* = 0.2672	r = −0.0890 *p* = 0.8496	r = 0.4474 *p* = 0.3141	r = −0.0698 *p* = 0.8818
TNF-α	r = 0.2669 *p* = 0.5628	r = 0.4940 *p* = 0.2598	r = 0.4033 *p* = 0.3696	r = −0.9310 * p = 0.0427

Note: * statistical difference; r = Pearson correlation coefficient.

**Table 4:** Correlation between urinary melatonin–sulfate and cytokines.

Melatonin–sulfate	NG-C	NG-UI	GDM-C	GDM-UI
IL-1 β	r = −0.1597 *p* = 0.7056	r = −0.1267 *p* = 0.7649	r = −0.2110 *p* = 0.6159	r = −0.4096 *p* = 0.3135
IL-6	r = −0.1461 *p* = 0.7298	r = 0.2588 *p* = 0.5360	r = 0.0018 *p* = 0.9966	r = 0.8984 * p = 0.0024
IL-8	r = 0.4240 *p* = 0.2941	r = 0.4627 *p* = 0.2482	r = −0.0991 *p* = 0.9829	r = 0.0278 *p* = 0.9480
IL-10	r = 0.3738 *p* = 0.3617	r = 0.1833 *p* = 0.6639	r = −0.6275 *p* = 0.0958	r = 0.7669 * p = 0.0263
IL-12	r = −0.0266 *p* = 0.9501	r = 0.1666 *p* = 0.8496	r = −0.2638 *p* = 0.5279	r = −0.4411 *p* = 0.2739
TNF-α	r = −0.2657 *p* = 0.5247	r = 0.0696 *p* = 0.6933	r = 0.2398 *p* = 0.5672	r = −0.5681 *p* = 0.1417

Note: * statistical difference; r = Pearson correlation coefficient.

## Data Availability

The data supporting the findings of this study are available from the corresponding authors upon reasonable request.

## References

[1] Moon J.H., Jang H.C. (2022). Gestational Diabetes Mellitus: Diagnostic Approaches and Maternal-Offspring Complications. Diabetes Metab. J..

[2] McIntyre H.D., Catalano P., Zhang C., Desoye G., Mathiesen E.R., Damm P. (2019). Gestational diabetes mellitus. Nat. Rev. Dis. Primers.

[3] Kim K.S., Hong S., Han K., Park C.Y. (2021). The clinical characteristics of gestational diabetes mellitus in Korea: A National Health Information Database Study. Endocrinol. Metab..

[4] Barbosa A.M.P., Enriquez E.M.A., Rodrigues M.R.K., Prudencio C.B., Atallah A.N., Reyes D.R.A., Hallur R.L.S., Nunes S.K., Pinheiro F.A., Filho C.I.S. (2020). Effectiveness of the pelvic floor muscle training on muscular dysfunction and pregnancy-specific urinary incontinence in pregnant women with gestational diabetes mellitus: A systematic review protocol. PLoS ONE.

[5] Huang H., Han X., Liu Q., Xue J., Yu Z., Miao S. (2022). Associations between metabolic syndrome and female stress urinary incontinence: A meta-analysis. Int. Urogynecol. J..

[6] Hirata Y., Nomura K., Senga Y., Okada Y., Kobayashi K., Okamoto S., Minokoshi Y., Imamura M., Takeda S., Hosooka T. (2019). Hyperglycemia induces skeletal muscle atrophy via a WWP1/KLF15 axis. JCI Insight.

[7] Reyes D.R.A., Barbosa A.M.P., Juliana F.F., Sofia Q.B.C.V., Costa S.M.B., Hallur R.L.S., Enriquez E.M.A., Oliveira R.G., Rossignolli P.S., Pedroni C.R. (2022). Viability of ex-vivo myography as a diagnostic tool for rectus abdominis muscle electrical activity collected at Cesarean section within a Diamater cohort study. Biomed. Eng. Online.

[8] Fagundes D.L.G., França E.L., Fernandes R.T.S., Hara C.C.P., Morceli G., Honorio-França A.C., Calderon I.M.P. (2016). Changes in T cell phenotype and cytokines profile in maternal blood, cord blood and colostrum of diabetic mothers. J. Matern. Fetal Neonatal Med..

[9] Kwon J., Kim D.Y., Cho K.J., Hashimoto M., Matsuoka K., Kamijo T., Wang Z., Karnup S., Robertson A.M., Tyagi P. (2024). Pathophysiology of overactive bladder and pharmacologic treatments including β3-adrenoceptor agonists—Basic research perspectives. Int. Neurourol. J..

[10] Tordjman S., Chokron S., Delorme R., Charrier A., Bellissant E., Jaafari N., Fougerou C. (2017). Melatonin: Pharmacology, functions and therapeutic benefits. Curr. Neuropharmacol..

[11] França D.C.H., Fujimori M., Queiroz A.A., Borges M.D., Magalhães Neto A.M., Camargos P.J.V., Ribeiro E.B., França E.L., Honorio-França A.C., Fagundes-Triches D.L.G. (2023). Melatonin and cytokines modulate daily instrumental activities of elderly people with SARS-CoV-2 infection. Int. J. Mol. Sci..

[12] França D.C.H., Honorio-França A.C., Silva K.M.R., Alves F.C.B., Bueno G., Costa S.M.B., Cotrim A.C.d.M., Barbosa A.M.P., França E.L., Rudge M.V.C. (2023). Serotonin and Interleukin-10 Can Influence Blood and Urine Viscosity in Gestational Diabetes Mellitus and Pregnancy-Specific Urinary Incontinence. Int. J. Mol. Sci..

[13] Ahmad S.B., Ali A., Bilal M., Rashid S.M., Wani A.B., Bhat R.R., Rehman M.U. (2023). Melatonin and health: Insights of melatonin action, biological functions, and associated disorders. Cell. Mol. Neurobiol..

[14] Hong Y., Jin Y., Park K., Choi J., Kang H., Lee S.-R., Hong Y. (2019). Elevated serum melatonin under constant darkness enhances neural repair in spinal cord injury through regulation of circadian clock proteins expression. J. Clin. Med..

[15] França D.C.H., Franca E.L., Sobrevia L., Barbosa A.M.P., Honorio-Franca A.C., Rudge M.V.C., Diamater Study Group (2023). Integration of nutrigenomics, melatonin, serotonin, and inflammatory cytokines in the pathophysiology of pregnancy-specific urinary incontinence in women with gestational diabetes mellitus. Biochim. Biophys. Acta Mol. Basis Dis..

[16] Veen A.V., Minović I., Faassen M.V., Gomes-Neto A.W., Berger S.P., Bakker S.J.L., Kema I.P. (2020). Urinary excretion of 6-sulfatoxymelatonin, the main metabolite of melatonin, and mortality in stable outpatient renal transplant recipients. J. Clin. Med..

[17] McMullan C.J., Schernhammer E.S., Rimm E.B., Hu F.B., Forman J.P. (2013). Melatonin secretion and the incidence of type 2 diabetes. JAMA.

[18] Aylamazyan E.K., Evsyukova I.I., Yarmolinskaya M.I. (2018). The role of melatonin in the development of gestational diabetes. MOJ Curr. Res. Rev..

[19] Ramsay S., Zagorodnyuk V. (2023). Role of circadian rhythms and melatonin in bladder function in health and diseases. Auton. Neurosci..

[20] Stenvers D.J., Scheer F.A.J.L., Schrauwen P., la Fleur S.E., Kalsbeek A. (2019). Circadian clocks and insulin resistance. Nat. Rev. Endocrinol..

[21] American Diabetes Association (2023). Standards of Medical Care in Diabetes—2023. Diabetes Care.

[22] Tamanini J.T.N., Dambros M., D’Ancona C.A.L., Palma P.C.R., Netto R. (2004). Validação para o português do “International Consultation on Incontinence Questionnaire-Short Form” (ICIQ-SF). Rev. Saude Publica.

[23] Pereira V.S., Santos J.Y.C., Correia G.N., Driusso P. (2011). Tradução e validação para a língua portuguesa de um questionário para avaliação da gravidade da incontinência urinária. Rev. Bras. Ginecol. Obstet..

[24] Muneeb H.N., Amjad M., Khaliq H.M., Shaukat K., Shabbir M., Shafique S., Hamid M.F. (2022). Association between pelvic floor dysfunction and metabolic syndrome: Pelvic floor dysfunction and metabolic syndrome. Pak. J. Med. Sci..

[25] Hardeland R. (2018). Melatonin and Inflammation—Story of a Double-Edged Blade. J. Pineal Res..

[26] Wang C., Jin L., Tong M., Zhang J., Yu J., Meng W., Jin L. (2022). Prevalence of gestational diabetes mellitus and its determinants among pregnant women in Beijing. J. Matern. Fetal Neonatal Med..

[27] Garmendia M.L., Mondschein S., Montiel B., Kusanovic J.P. (2020). Trends and predictors of gestational diabetes mellitus in Chile. Int. J. Gynaecol. Obstet..

[28] Cornejo M., Fuentes G., Valero P., Vega S., Grismaldo A., Toledo F., Pardo F., Moore-Carrasco R., Subiabre M., Casanello P. (2021). Gestational diabesity and foetoplacental vascular dysfunction. Acta Physiol..

[29] Plows J.F., Stanley J.L., Baker P.N., Reynolds C.M., Vickers M.H. (2018). The pathophysiology of gestational diabetes mellitus. Int. J. Mol. Sci..

[30] Morceli G., Honorio-França A.C., Fagundes D.L.G., Calderon I.M.P., França E.L. (2013). Antioxidant Effect of Melatonin on the Functional Activity of Colostral Phagocytes in Diabetic Women. PLoS ONE.

[31] Mendes L., Queiroz M., Sena C.M. (2024). Melatonin and Vascular Function. Antioxidants.

[32] Pozo M.J., Gomez-Pinilla P.J., Camello-Almaraz C., Martin-Cano F.E., Pascua P., Rol M.A., Acuna-Castroviejo D., Camello P.J. (2010). Melatonin, a Potential Therapeutic Agent for Smooth Muscle-Related Pathological Conditions and Aging. Curr. Med. Chem..

[33] Hanna-Mitchel A.T., Robinson D., Cardozo L., Everaert K., Petkov G.V. (2016). Do We Need to Know More about the Effects of Hormones on Lower Urinary Tract Dysfunction? ICI-RS 2014. Neurourol. Urodyn..

[34] Gomez-Pinilla P.J., Pozo M.J., Camello P.J. (2007). Aging Impairs Neurogenic Contraction in Guinea Pig Urinary Bladder: Role of Oxidative Stress and Melatonin. Am. J. Physiol. Regul. Integr. Comp. Physiol..

[35] Koppisetti S., Jenigiri B., Terron M.P., Tengattini S., Tamura H., Flores L.J., Tan D.X., Reiter R.J. (2008). Reactive Oxygen Species and the Hypomotility of the Gall Bladder as Targets for the Treatment of Gallstones with Melatonin: A Review. Dig. Dis. Sci..

[36] Gomez-Pinilla P.J., Gomez M.F., Sward K., Hedlund P., Hellstrand P., Camello P.J., Andersson K.E., Pozo M.J. (2008). Melatonin Restores Impaired Contractility in Aged Guinea Pig Urinary Bladder. J. Pineal Res..

[37] Owino S., Sánchez-Bretaño A., Tchio C., Cecon E., Karamitri A., Dam J., Jockers R., Piccione G., Noh H.L., Kim T. (2018). Nocturnal Activation of Melatonin Receptor Type 1 Signaling Modulates Diurnal Insulin Sensitivity via Regulation of PI3K Activity. J. Pineal Res..

[38] Owino S., Buonfiglio D.D.C., Tchio C., Tosini G. (2019). Melatonin Signaling: A Key Regulator of Glucose Homeostasis and Energy Metabolism. Front. Endocrinol..

[39] Carlomagno G., Minini M., Tilotta M., Unfer V. (2018). From Implantation to Birth: Insight into Molecular Melatonin Functions. Int. J. Mol. Sci..

[40] Man G.C.W., Zhang T., Chen X., Wang J., Wu F., Liu Y., Wang C.C., Cheong Y., Li T.C. (2017). The Regulations and Role of Circadian Clock and Melatonin in Uterine Receptivity and Pregnancy—An Immunological Perspective. Am. J. Reprod. Immunol..

[41] Haroon E., Raison C.L., Miller A.H. (2012). Psychoneuroimmunology Meets Neuropsychopharmacology: Translational Implications of the Impact of Inflammation on Behavior. Neuropsychopharmacology.

[42] Chess-Williams R., McDermott C., Sellers D.J., West E.G., Mills K.A. (2021). Chronic Psychological Stress and Lower Urinary Tract Symptoms. Low. Urin. Tract Symptoms.

[43] Jiang Y.H., Jhang J.F., Hsu Y.H., Ho H.C., Wu Y.H., Kuo H.C. (2020). Urine Cytokines as Biomarkers for Diagnosing Interstitial Cystitis/Bladder Pain Syndrome and Mapping Its Clinical Characteristics. Am. J. Physiol. Renal Physiol..

